# Three new species of the *Stenus cirrus* group from Guizhou, southwest China (Coleoptera, Staphylinidae)

**DOI:** 10.3897/zookeys.716.20619

**Published:** 2017-11-27

**Authors:** Sheng-Nan Liu, Liang Tang, Yong-Ting Luo

**Affiliations:** 1 Department of Biology, Shanghai Normal University, 100 Guilin Road, 1st Educational Building 323 Room, Shanghai, 200234 P. R. China

**Keywords:** China, Coleoptera, Guizhou, new species, Staphylinidae, *Stenus cirrus* group

## Abstract

Three new *Stenus* species of the *cirrus* group collected from Guizhou Province, southwest China, are described: *S. dashaheensis* **sp. n.**, *S. zhangyuqingi* **sp. n.**, and *S. liuyixiaoi* **sp. n.** The diagnostic characters of the new species are illustrated, and a key to species of the group from Guizhou Province is provided.

## Introduction

The *Stenus cirrus* group is a large group with 75 known species worldwide, 58 of which have been reported from China (Tang et al. 2008; Puthz 2009; Tang et al. 2016; Liu et al. 2017). The group was hitherto not recorded from Guizhou province, but three new species were collected recently and are described in this paper.

## Materials and methods

The specimens examined in this paper were mainly collected at various locations in Guizhou, southwest China, by sifting leaf litter in broad-leaved forests. Specimens were euthanized with ethyl acetate and dried. For examination of the male and female genitalia, the apical three abdominal segments were detached from the body after softening in hot water. The aedeagi, together with other dissected parts, were mounted in Euparal (Chroma Gesellschaft Schmidt, Koengen, Germany) on plastic slides. Photographs of genitalia were taken with a Canon G9 camera attached to an Olympus CX31 microscope; habitus photos were taken with a Canon macro photo lens MP-E 65 mm attached to a Canon EOS7D camera and stacked with Zerene Stacker.

The type specimens treated in this study are deposited in the following public and private collections:

**SHNU** Department of Biology, Shanghai Normal University, P. R. China;

**cPut** Private collection V. Puthz, Schlitz, Germany.

The measurements of proportions are abbreviated as follows:

**BL** body length, measured from the anterior margin of the clypeus to the posterior margin of abdominal tergite X;

**FL** fore-body length, measured from the anterior margin of the clypeus to the apicolateral angle of elytra;

**HW** width of head including eyes;

**PW** width of pronotum;

**EW** width of elytra;

**PL** length of pronotum;

**EL** length of elytra, measured from humeral angle;

**SL** length of elytral suture.

## Taxonomy

### Stenus dashaheensis sp. n.

Taxon classificationAnimaliaColeopteraStaphylinidae

http://zoobank.org/F2CE4CFE-607D-40E0-A6AA-E31D7CAE65B1

Figs 1 , 2 , 7–12

#### Material examined.

**CHINA: Guizhou: Holotype**: ♂, glued on a card with labels as follows: “China: N. Guizhou, Daozhen Co., Dashahe, 29°10'12"N, 107°33'36"E, mixed leaf litter, sifted, 1730 m, 07.VII.2015, Jiang, Peng, Tu & Zhou leg.”. “Holotype / *Stenus dashaheensis* / Liu, Tang & Luo” [red handwritten label] (SHNU). **Paratypes**: 2♂♂3♀♀, same data as for the holotype. (SHNU, cPut).

**Figures 1–6.**
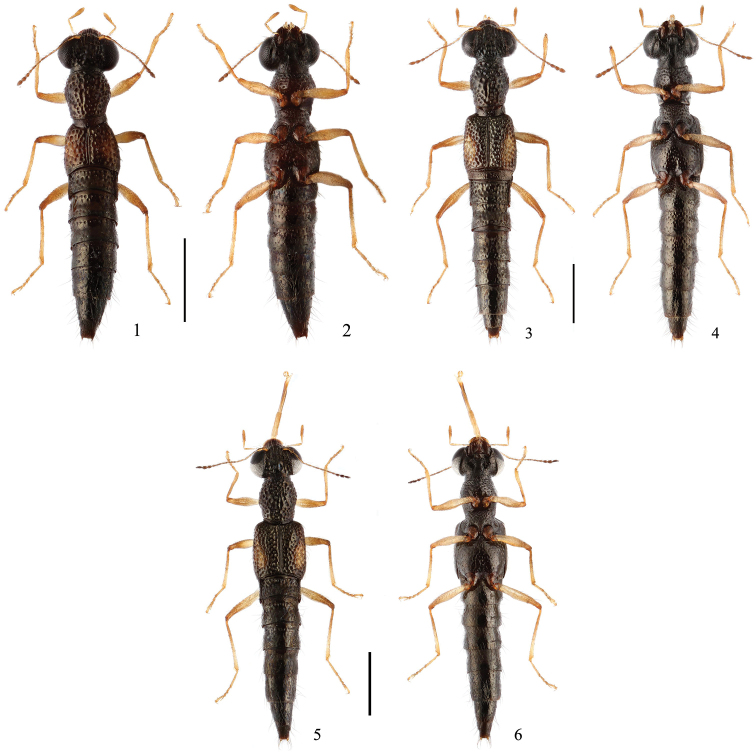
Habitus. **1, 2** *Stenus dashaheensis* sp. n. **3, 4** *Stenus zhangyuqingi* sp. n. **5, 6** *Stenus liuyixiaoi* sp. n. Scale bars: 1 mm.

#### Diagnosis.

The new species can be distinguished from other related species by the following characters: body size smaller (BL < 4.2 mm), elytra distinctly shorter than wide (EL/EW= 0.84–0.90), the rather smooth surfaces of pronotum and elytra, and the larger and less confluent punctation of pronotum and elytra than abdomen.

#### Description.

Brachypterous; body dark brown, each elytron with an orange spot near lateral margin. Antennae except the infuscate club, maxillary palpi, and legs yellowish brown. Labrum reddish brown. BL: 3.3–4.1 mm, FL: 1.7–2.0 mm. HW: 0.69–0.84 mm, PL: 0.55–0.65 mm, PW: 0.53–0.61 mm, EL: 0.57–0.68 mm, EW: 0.64–0.78 mm, SL: 0.43–0.49 mm.

*Head* 1.06–1.18 times as wide as elytra; interocular area with two deep longitudinal furrows, median portion convex, extending beneath the level of inner eye margins; punctures round, slightly larger and sparser on median portion than those near inner margins of eyes, diameter of large punctures slightly wider than apical cross section of antennal segment II; interstices between punctures smooth, much narrower than half the diameter of punctures except those along the midline of the median portion, which may be slightly narrower than the diameter of punctures. Paraglossae oval.

*Pronotum* 0.98–1.05 times as long as wide; disk relatively smooth (without impressions) with median longitudinal furrow indistinct; punctures round and slightly confluent, variable in size, slightly larger than those of head; interstices smooth, much narrower than half the diameter of punctures except for few near the actual middle, which may be as wide as the diameter of punctures.

*Elytra* 0.84–0.90 times as long as wide; disk relatively smooth; punctures round to elliptical, moderately confluent, similar in size to those on pronotum; interstices smooth, distinctly smaller than half the diameter of punctures.

*Legs* with tarsomeres IV strongly bilobed.

*Abdomen* cylindrical; paratergites very narrow and almost impunctate, present only in segment III, tergites and sternites totally fused in segments IV–VI, posterior margin of tergite VII with an indistinct apical membranous fringe; punctation of tergites III–VIII sparse and shallow, gradually becoming smaller posteriorly; interstices smooth, mostly wider than diameter of punctures except those on basal impressions of tergites III–V, which may be distinctly narrower than half the diameter of punctures.

**Figures 7–12.**
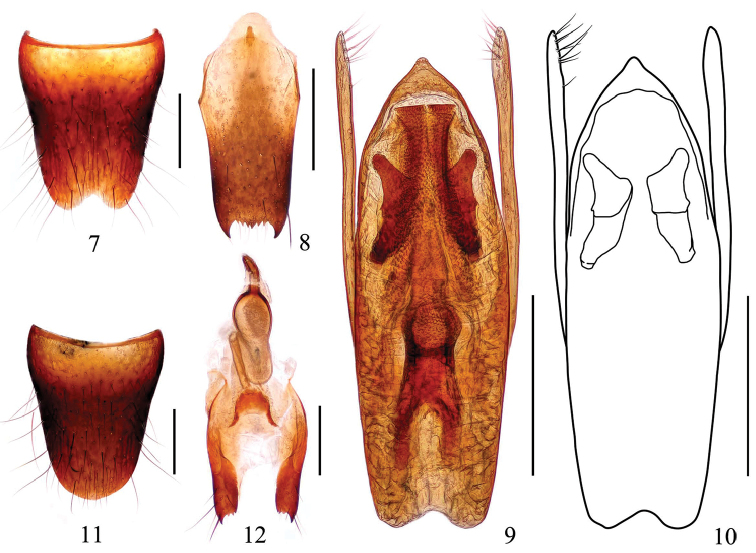
*Stenus dashaheensis* sp. n. **7** male sternite VIII **8** male sternite IX **9, 10** aedeagus.**11** female sternite VIII **12** valvifers and spermatheca. Scale bars: 0.25 mm.

Male. Sternite VIII (Fig. 7) with distinct triangular emargination at middle of posterior margin; sternite IX (Fig. 8) with long apicolateral projections, posterior margin serrated. Aedeagus (Figs 9–10) with apical sclerotized portion weakly prominent at apex; sclerotized expulsion clasps large, median ventral band long, narrow, dorsal bands short and relatively broad, lateral longitudinal bands short; copulatory tube stout; parameres longer than median lobe, each with 10–13 setae on apico-internal margins.

Female. Sternite VIII as in Fig. 11; sclerotized spermatheca (Fig. 12) consisting of basal duct, swollen spermathecal duct, and capsule.

#### Distribution.

China (Guizhou).

#### Remarks.

The new species is similar to *S. bullatus* Liu & Tang, 2017 from Guangxi, but can be easily distinguished from the latter by the less confluent punctation of the pronotum and the elytra, and by shorter elytra (in *S. bullatus* EL/EW = 0.90–1.07).

#### Etymology.

The specific name is derived from the type locality of this species.

### Stenus zhangyuqingi sp. n.

Taxon classificationAnimaliaColeopteraStaphylinidae

http://zoobank.org/44875E61-1CBA-4D9E-8A7E-8E47ABE1526F

Figs 3 , 4 , 13–18

#### Material examined.

**CHINA: Guizhou: Holotype**: ♂, glued on a card with labels as follows: “China: N. Guizhou, Libo County Maolan N.R., Dongdai, 25°17'13"N, 107°56'23"E, 792 m, 24.iv.2017, mixed leaf litter, sifted, Jiang, Jiang, Hu, Liu & Zhang leg.”. “Holotype / *Stenus zhangyuqingi* / Liu, Tang & Luo” [red handwritten label]. **Paratypes**: 4♂♂5♀♀, same data as for the holotype. (SHNU, cPut).

#### Diagnosis.

The new species is the most characteristic of the group characterized by the largest body, longer than 4.2 mm, a broad head up to 0.83–0.96 mm, and a shallower and narrower median longitudinal furrow of the pronotum than the other two species in this paper.

#### Description.

Macropterous; body blackish, each elytron with an elongated yellow spot near lateral margin. Antennae (except the infuscate club), maxillary palpi, and legs yellowish brown. Labrum reddish brown. BL: 4.2–5.1 mm, FL: 2.1–2.5 mm. HW: 0.83–0.96 mm, PL: 0.64–0.79 mm, PW: 0.61–0.69 mm, EL: 0.84–1.00 mm, EW: 0.78–0.93 mm, SL: 0.68–0.79 mm.

*Head* 1.03–1.13 times as wide as elytra; interocular area with two deep longitudinal furrows, median portion convex, extending beneath the level of inner eye margins; punctures round and more or less confluent, larger and sparser on median portion than those near inner margins of eyes, diameter of large punctures larger than apical cross-section of antennal segment II; interstices between punctures smooth, much narrower than half the diameter of punctures except those along the midline of the median portion, which may be as wide as half the diameter of punctures. Paraglossae oval.

*Pronotum* 1.02–1.15 times as long as wide; disk with impressions, with a shallow and narrow median longitudinal furrow; punctures round and moderately confluent, variable in size, on average larger than those of head; interstices smooth, much narrower than half the diameter of punctures except for those along the middle of posterior half pronotum, which may be as wide as the diameter of punctures.

*Elytra* 1.07–1.16 times as long as wide; disk smooth; punctures round, moderately confluent, slightly larger than those on pronotum; interstices smooth, distinctly smaller than half the diameter of punctures.

*Legs* with tarsomeres IV strongly bilobed.

*Abdomen* cylindrical; paratergites very narrow and almost impunctate, visible only in segment III, tergites and sternites totally fused in segments IV–VI, posterior margin of tergite VII with apical membranous fringe; punctation of tergites III–VIII sparse and shallow, gradually becoming smaller posteriorly; interstices smooth, narrower than half the diameter of punctures on tergite III, narrower than half the diameter to diameter of punctures on tergites III and IV.

**Figures 13–18.**
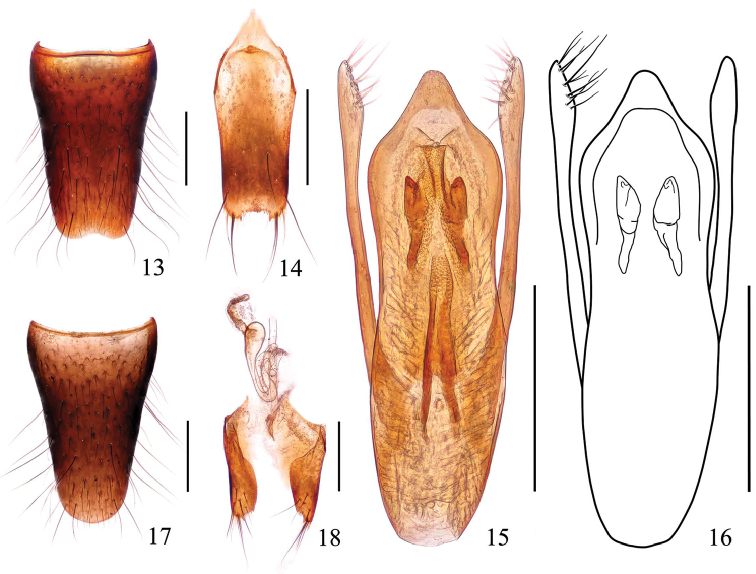
*Stenus zhangyuqingi* sp. n. **13** male sternite VIII **14** male sternite IX **15, 16** aedeagus **17** female sternite VIII **18** valvifers and spermatheca. Scale bars: 0.25 mm.

Male. Sternite VIII (Fig. 13) with shallow emargination at middle of posterior margin; sternite IX (Fig. 14) with long apicolateral projections, posterior margin serrate. Aedeagus (Figs 15–16) with apical sclerotized portion triangular , convex at apex; internal structures: sclerotized expulsion clasps long, median ventral band long, narrow, dorsal bands long, lateral bands short; copulatory tube rather short, the main tube weakly curved near the middle; parameres longer than median lobe, slightly swollen in apical part, each with 10–12 setae on apicointernal margins.

Female. Sternite VIII as in Fig. 17; sclerotized spermatheca (Fig. 18) with spermathecal duct very coiled.

#### Distribution.

China (Guizhou).

#### Remarks.

The new species is closely related to *S. guangxiensis* Rougemont, 1984 from Zhejiang, Anhui, and Guangxi, as well as to *S. liuyixiaoi* sp. n., but can be easily distinguished from them by the narrower elytra (in the other two species, HW similar to or distinctly smaller than EW) and different sexual characters.

#### Etymology.

This species is named in honor of Mr. Yu-Qing Zhang who collected some of the specimens of the new species.

### Stenus liuyixiaoi sp. n.

Taxon classificationAnimaliaColeopteraStaphylinidae

http://zoobank.org/64D47988-137C-47E8-9786-1FF1A31BAD3E

Figs 5 , 6 , 19–24

#### Material examined.

**CHINA: Guizhou: Holotype**: ♂, glued on a card with labels as follows: “China: N. Guizhou, Libo County Maolan N.R., Bizuo, 25°16'59"N, 106°03'18"E, 587 m, 28.iv.2017, mixed leaf litter, sifted, Jiang, Jiang, Hu, Liu & Zhang leg.”. “Holotype / *Stenus liuyixiaoi* / Liu, Tang & Luo” [red handwritten label] (SHNU). **Paratypes**: 6♂♂9♀♀, same data as for the holotype (SHNU, cPut); 2♀♀, Maolan N.R., Dongdai, 25°17'51"N, 107°57'15"E, 808 m, 22.iv.2017, mixed leaf litter, sifted, Jiang, Jiang, Hu, Liu & Zhang leg. (SHNU); 4♂♂3♀♀, Maolan N.R., Dongdai, 25°17'58"N, 107°57'06"E, 874 m, 25.iv.2017, mixed leaf litter, sifted, Jiang, Jiang, Hu, Liu & Zhang leg. (SHNU); 1♂2♀♀, Dongdai, 25°17'13"N, 107°56'23"E, 792 m, 24.iv.2017, mixed leaf litter, sifted, Jiang, Jiang, Hu, Liu & Zhang leg. (SHNU).

#### Diagnosis.

The new species is characterized by large body size (4.2–5.8 mm) and relatively sparse punctation of the entire body, especially of the abdominal tergites.

#### Description.

Macropterous; body blackish, each elytron with a large yellow spot near its lateral margin. Antennae (except the infuscate club), maxillary palpi, and legs yellowish brown. Labrum reddish brown. BL: 4.2–5.8 mm, FL: 2.0–2.6 mm. HW: 0.77–0.96 mm, PL: 0.64–0.78 mm, PW: 0.61–0.72 mm, EL: 0.90–1.07 mm, EW: 0.82–1.01 mm, SL: 0.72–0.86 mm.

*Head* 0.93–1.03 times as wide as elytra, pronotum 1.06–1.13 times as long as wide, elytra 1.03–1.12 times as long as wide.

Other external characters as in *S. zhangyuqingi* sp. n., except that the punctation of the pronotum and abdomen is slightly sparser.

**Figures 19–24.**
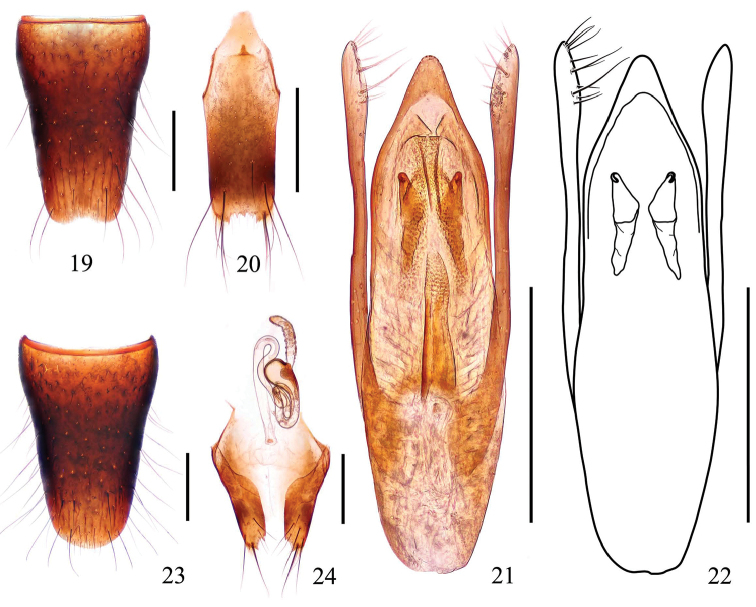
*Stenus liuyixiaoi* sp. n. **19** male sternite VIII **20** male sternite IX **21, 22** aedeagus **23** female sternite VIII **24** valvifers and spermatheca. Scale bars: 0.25 mm.

Male. Sternite VIII (Fig. 19) with shallow emargination at middle of posterior margin; sternite IX (Fig. 20) with long apicolateral projections, posterior margin serrated. Aedeagus (Figs 21–22) with triangular apical sclerotized portion and convex apex; internal structures: sclerotized expulsion clasps large, median ventral band long, narrow, lateral bands short; copulatory tube long, weakly curved in the middle; parameres longer than median lobe, swollen at apical parts, each with 11–13 setae on apicointernal margins.

Female. Sternite VIII as in Fig. 23; spermatheca (Fig. 24) strongly sclerotized with

spermathecal duct very coiled.

**Distribution.** China (Guizhou).

**Remarks.** The new species is closely related to *S. zhangyuqingi* sp. n., but may be distinguished from the latter by the relatively sparser punctation of pronotum and abdomen. It is also very similar to *S. guangxiensis* Rougemont, 1984, from which it is distinguished only based on the sexual characters.

**Etymology.** This species is named in honor of Mr. Yi-Xiao Liu who collected some specimens of the new species.

##### Key to species of the *Stenus cirrus* group of Guizhou

**Table:** 

1	Brachypterous, elytra distinctly shorter than wide. Habitus: Figs 1, 2; sexual characters: Figs 7–12. BL: 3.3–4.1 mm	***S. dashaheensis* sp. n.**
–	Macropterous, elytra distinctly longer than wide	2
2	Elytra broader with HW/EW = 0.93–1.03. Habitus: Figs 5, 6; sexual characters: Figs 19–24. BL: 4.2–5.8 mm	***S. liuyixiaoi* sp. n.**
–	Elytra narrower with HW/EW = 1.03–1.13. Habitus: Figs 3, 4; sexual characters: Figs 13–18. BL: 4.2–5.1 mm	***S. zhangyuqingi* sp. n.**

## Supplementary Material

XML Treatment for Stenus dashaheensis

XML Treatment for Stenus zhangyuqingi

XML Treatment for Stenus liuyixiaoi
